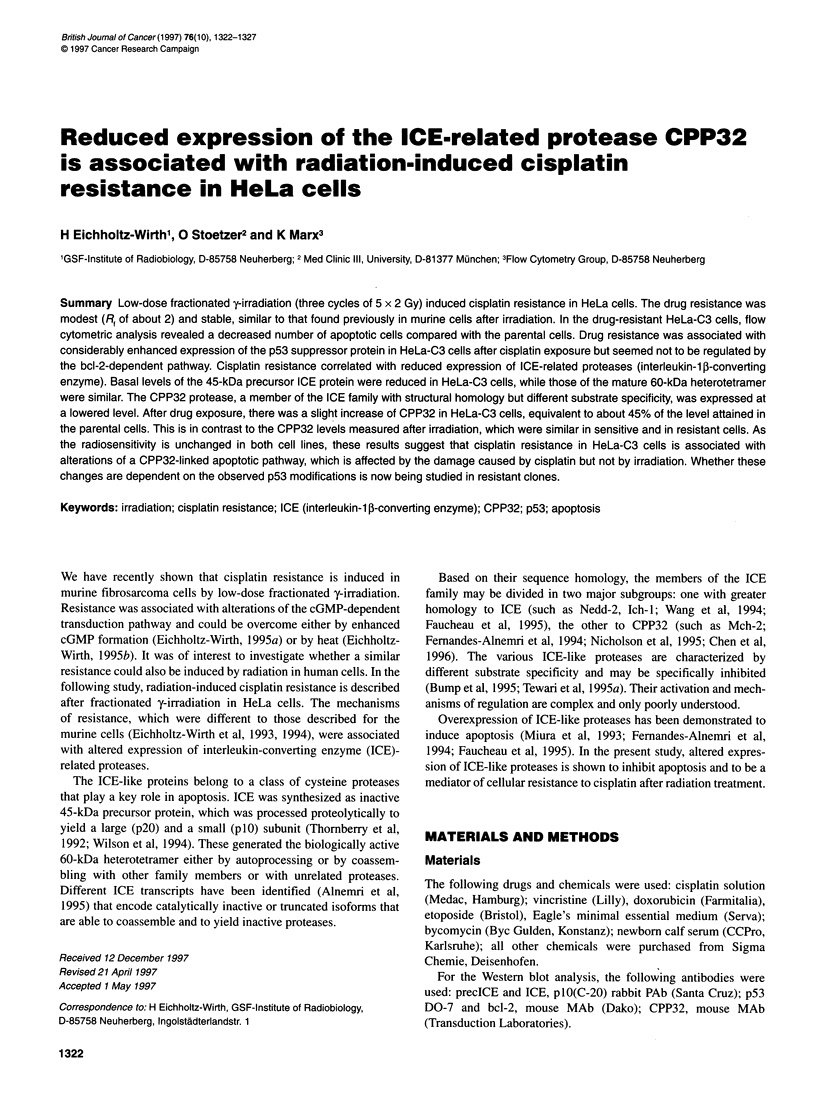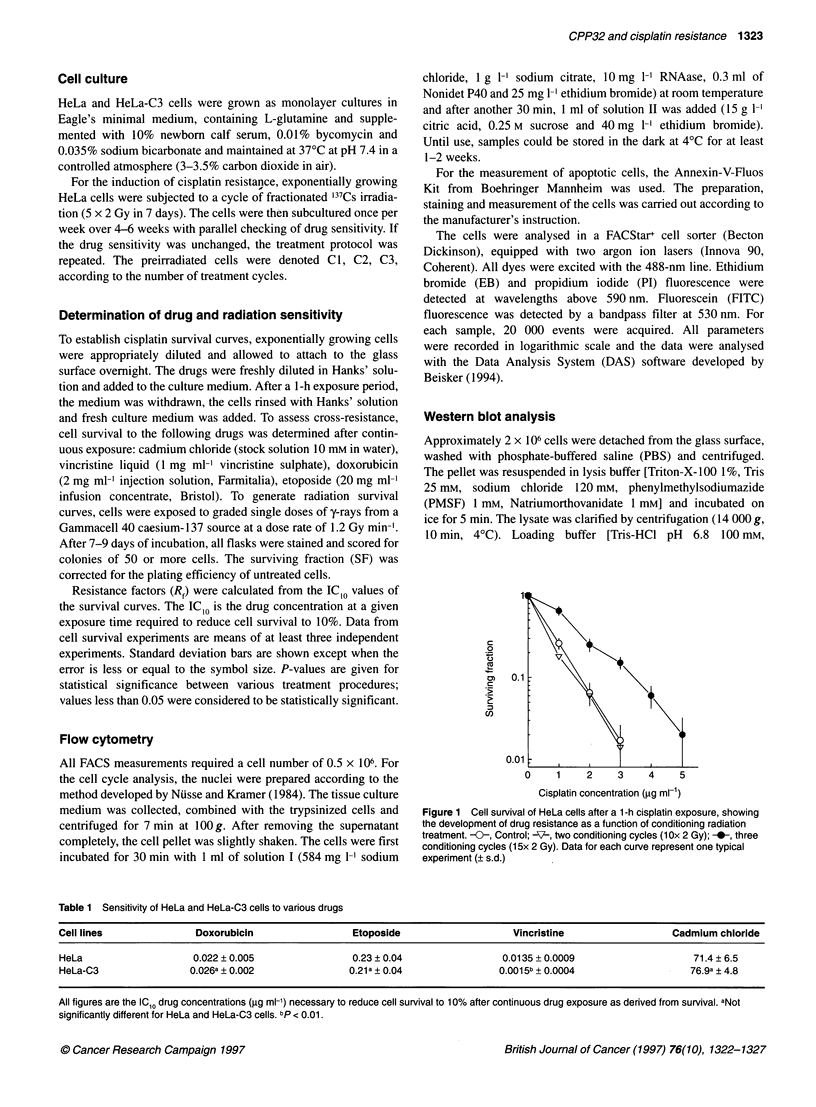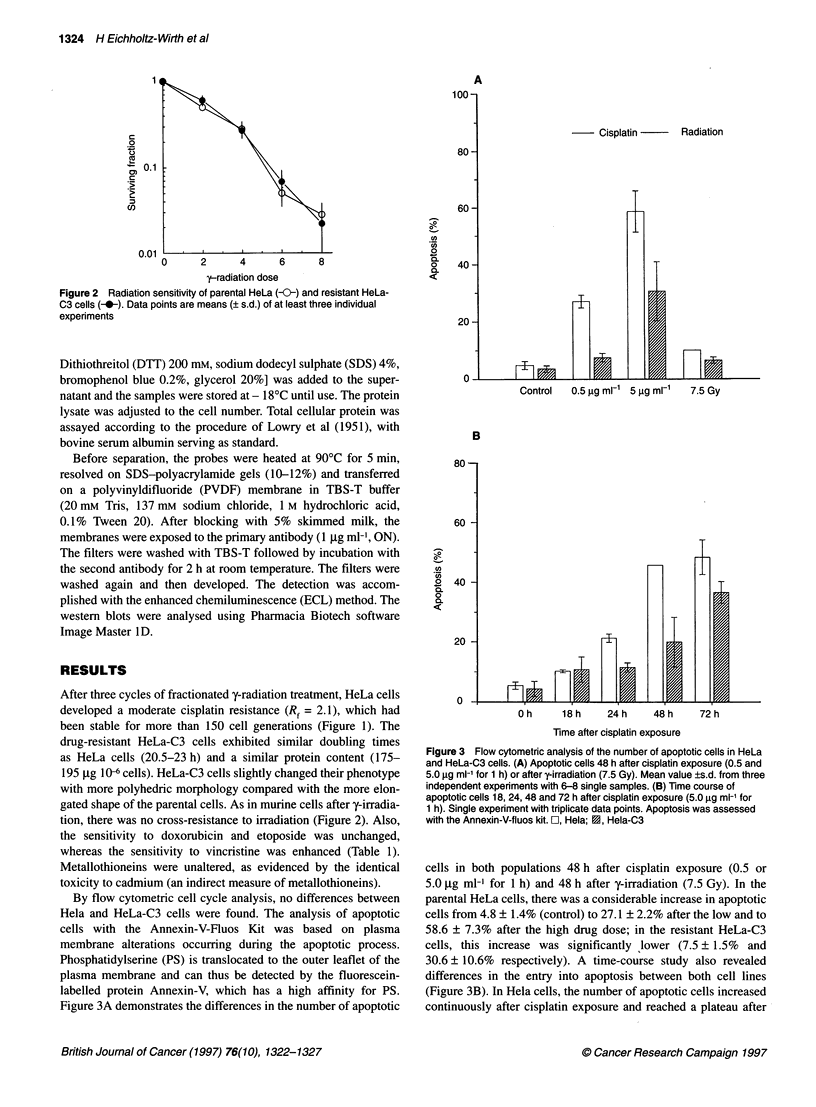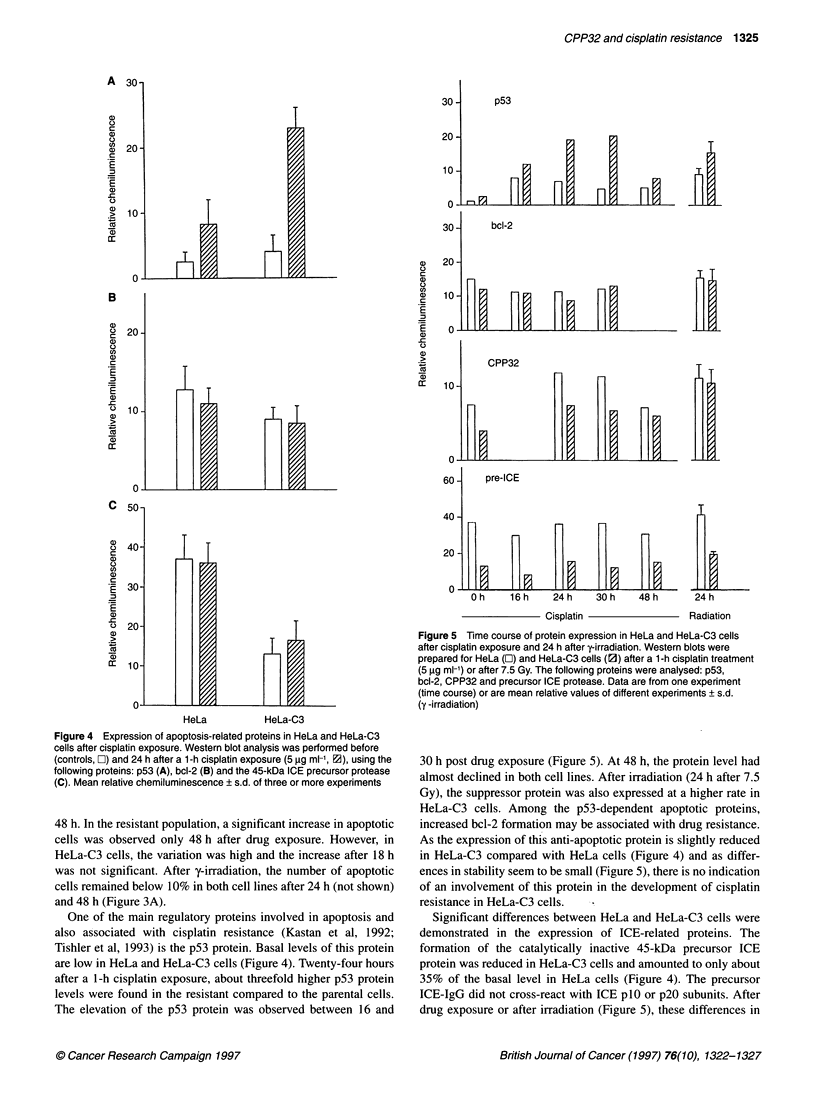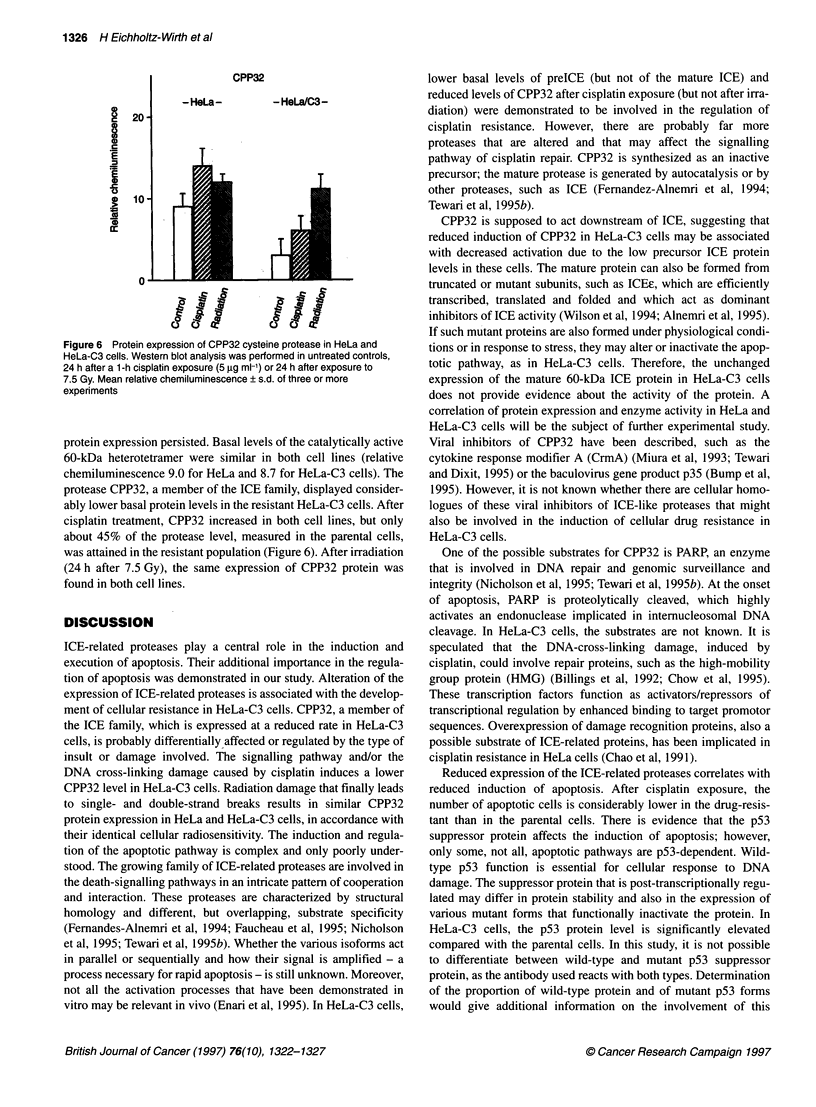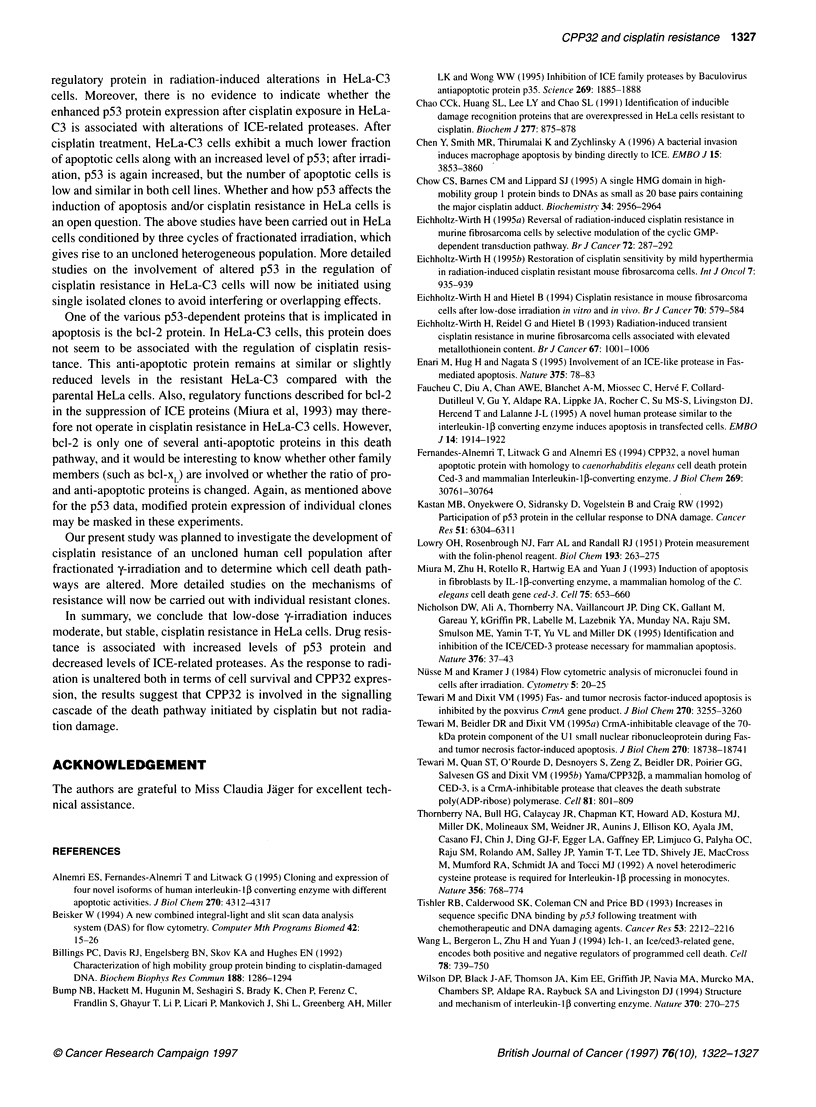# Reduced expression of the ICE-related protease CPP32 is associated with radiation-induced cisplatin resistance in HeLa cells.

**DOI:** 10.1038/bjc.1997.555

**Published:** 1997

**Authors:** H. Eichholtz-Wirth, O. Stoetzer, K. Marx

**Affiliations:** GSF-Institute of Radiobiology, Neuherberg.

## Abstract

Low-dose fractionated gamma-irradiation (three cycles of 5 x 2 Gy) induced cisplatin resistance in HeLa cells. The drug resistance was modest (Rf of about 2) and stable, similar to that found previously in murine cells after irradiation. In the drug-resistant HeLa-C3 cells, flow cytometric analysis revealed a decreased number of apoptotic cells compared with the parental cells. Drug resistance was associated with considerably enhanced expression of the p53 suppressor protein in HeLa-C3 cells after cisplatin exposure but seemed not to be regulated by the bcl-2-dependent pathway. Cisplatin resistance correlated with reduced expression of ICE-related proteases (interleukin-1beta-converting enzyme). Basal levels of the 45-kDa precursor ICE protein were reduced in HeLa-C3 cells, while those of the mature 60-kDa heterotetramer were similar. The CPP32 protease, a member of the ICE family with structural homology but different substrate specificity, was expressed at a lowered level. After drug exposure, there was a slight increase of CPP32 in HeLa-C3 cells, equivalent to about 45% of the level attained in the parental cells. This is in contrast to the CPP32 levels measured after irradiation, which were similar in sensitive and in resistant cells. As the radiosensitivity is unchanged in both cell lines, these results suggest that cisplatin resistance in HeLa-C3 cells is associated with alterations of a CPP32-linked apoptotic pathway, which is affected by the damage caused by cisplatin but not by irradiation. Whether these changes are dependent on the observed p53 modifications is now being studied in resistant clones.


British Journal of Cancer (1997) 76(10), 1322–1327
© 1997 Cancer Research Campaign

### Reduced expression of the ICE-related protease CPP32 is associated with radiation-induced cisplatin resistance in HeLa cells

**H Eichholtz-Wirth[1], O Stoetzer[2] and K Marx[3]**

[1]GSF-Institute of Radiobiology, D-85758 Neuherberg; [2] Med Clinic III, University, D-81377 München; [3]Flow Cytometry Group, D-85758 Neuherberg

**Summary** Low-dose fractionated γ-irradiation (three cycles of 5 × 2 Gy) induced cisplatin resistance in HeLa cells. The drug resistance was modest ($R_f$ of about 2) and stable, similar to that found previously in murine cells after irradiation. In the drug-resistant HeLa-C3 cells, flow cytometric analysis revealed a decreased number of apoptotic cells compared with the parental cells. Drug resistance was associated with considerably enhanced expression of the p53 suppressor protein in HeLa-C3 cells after cisplatin exposure but seemed not to be regulated by the bcl-2-dependent pathway. Cisplatin resistance correlated with reduced expression of ICE-related proteases (interleukin-1β-converting enzyme). Basal levels of the 45-kDa precursor ICE protein were reduced in HeLa-C3 cells, while those of the mature 60-kDa heterotetramer were similar. The CPP32 protease, a member of the ICE family with structural homology but different substrate specificity, was expressed at a lowered level. After drug exposure, there was a slight increase of CPP32 in HeLa-C3 cells, equivalent to about 45% of the level attained in the parental cells. This is in contrast to the CPP32 levels measured after irradiation, which were similar in sensitive and in resistant cells. As the radiosensitivity is unchanged in both cell lines, these results suggest that cisplatin resistance in HeLa-C3 cells is associated with alterations of a CPP32-linked apoptotic pathway, which is affected by the damage caused by cisplatin but not by irradiation. Whether these changes are dependent on the observed p53 modifications is now being studied in resistant clones.

**Keywords:** irradiation; cisplatin resistance; ICE (interleukin-1β-converting enzyme); CPP32; p53; apoptosis

We have recently shown that cisplatin resistance is induced in murine fibrosarcoma cells by low-dose fractionated γ-irradiation. Resistance was associated with alterations of the cGMP-dependent transduction pathway and could be overcome either by enhanced cGMP formation (Eichholtz-Wirth, 1995*a*) or by heat (Eichholtz-Wirth, 1995*b*). It was of interest to investigate whether a similar resistance could also be induced by radiation in human cells. In the following study, radiation-induced cisplatin resistance is described after fractionated γ-irradiation in HeLa cells. The mechanisms of resistance, which were different to those described for the murine cells (Eichholtz-Wirth et al, 1993, 1994), were associated with altered expression of interleukin-converting enzyme (ICE)-related proteases.

The ICE-like proteins belong to a class of cysteine proteases that play a key role in apoptosis. ICE was synthesized as inactive 45-kDa precursor protein, which was processed proteolytically to yield a large (p20) and a small (p10) subunit (Thornberry et al, 1992; Wilson et al, 1994). These generated the biologically active 60-kDa heterotetramer either by autoprocessing or by coassembling with other family members or with unrelated proteases. Different ICE transcripts have been identified (Alnemri et al, 1995) that encode catalytically inactive or truncated isoforms that are able to coassemble and to yield inactive proteases.

*Received 12 December 1997*
*Revised 21 April 1997*
*Accepted 1 May 1997*

*Correspondence to:* H Eichholtz-Wirth, GSF-Institute of Radiobiology, D-85758 Neuherberg, Ingolstädterlandstr. 1

Based on their sequence homology, the members of the ICE family may be divided in two major subgroups: one with greater homology to ICE (such as Nedd-2, Ich-1; Wang et al, 1994; Faucheau et al, 1995), the other to CPP32 (such as Mch-2; Fernandes-Alnemri et al, 1994; Nicholson et al, 1995; Chen et al, 1996). The various ICE-like proteases are characterized by different substrate specificity and may be specifically inhibited (Bump et al, 1995; Tewari et al, 1995*a*). Their activation and mechanisms of regulation are complex and only poorly understood.

Overexpression of ICE-like proteases has been demonstrated to induce apoptosis (Miura et al, 1993; Fernandes-Alnemri et al, 1994; Faucheau et al, 1995). In the present study, altered expression of ICE-like proteases is shown to inhibit apoptosis and to be a mediator of cellular resistance to cisplatin after radiation treatment.

### MATERIALS AND METHODS

### Materials

The following drugs and chemicals were used: cisplatin solution (Medac, Hamburg); vincristine (Lilly), doxorubicin (Farmitalia), etoposide (Bristol), Eagle's minimal essential medium (Serva); bycomycin (Byc Gulden, Konstanz); newborn calf serum (CCPro, Karlsruhe); all other chemicals were purchased from Sigma Chemie, Deisenhofen.

For the Western blot analysis, the following antibodies were used: precICE and ICE, p10(C-20) rabbit PAb (Santa Cruz); p53 DO-7 and bcl-2, mouse MAb (Dako); CPP32, mouse MAb (Transduction Laboratories).

### Cell culture

HeLa and HeLa-C3 cells were grown as monolayer cultures in Eagle's minimal medium, containing L-glutamine and supplemented with 10% newborn calf serum, 0.01% bycomycin and 0.035% sodium bicarbonate and maintained at 37°C at pH 7.4 in a controlled atmosphere (3–3.5% carbon dioxide in air).

For the induction of cisplatin resistance, exponentially growing HeLa cells were subjected to a cycle of fractionated $^{137}Cs$ irradiation (5 × 2 Gy in 7 days). The cells were then subcultured once per week over 4–6 weeks with parallel checking of drug sensitivity. If the drug sensitivity was unchanged, the treatment protocol was repeated. The preirradiated cells were denoted C1, C2, C3, according to the number of treatment cycles.

### Determination of drug and radiation sensitivity

To establish cisplatin survival curves, exponentially growing cells were appropriately diluted and allowed to attach to the glass surface overnight. The drugs were freshly diluted in Hanks' solution and added to the culture medium. After a 1-h exposure period, the medium was withdrawn, the cells rinsed with Hanks' solution and fresh culture medium was added. To assess cross-resistance, cell survival to the following drugs was determined after continuous exposure: cadmium chloride (stock solution 10 mM in water), vincristine liquid (1 mg $ml^{-1}$ vincristine sulphate), doxorubicin (2 mg $ml^{-1}$ injection solution, Farmitalia), etoposide (20 mg $ml^{-1}$ infusion concentrate, Bristol). To generate radiation survival curves, cells were exposed to graded single doses of γ-rays from a Gammacell 40 caesium-137 source at a dose rate of 1.2 Gy $min^{-1}$. After 7–9 days of incubation, all flasks were stained and scored for colonies of 50 or more cells. The surviving fraction (SF) was corrected for the plating efficiency of untreated cells.

Resistance factors ($R_f$) were calculated from the $IC_{10}$ values of the survival curves. The $IC_{10}$ is the drug concentration at a given exposure time required to reduce cell survival to 10%. Data from cell survival experiments are means of at least three independent experiments. Standard deviation bars are shown except when the error is less or equal to the symbol size. $P$-values are given for statistical significance between various treatment procedures; values less than 0.05 were considered to be statistically significant.

### Flow cytometry

All FACS measurements required a cell number of 0.5 × $10^6$. For the cell cycle analysis, the nuclei were prepared according to the method developed by Nüsse and Kramer (1984). The tissue culture medium was collected, combined with the trypsinized cells and centrifuged for 7 min at 100 *g*. After removing the supernatant completely, the cell pellet was slightly shaken. The cells were first incubated for 30 min with 1 ml of solution I (584 mg $l^{-1}$ sodium chloride, 1 g $l^{-1}$ sodium citrate, 10 mg $l^{-1}$ RNAase, 0.3 ml of Nonidet P40 and 25 mg $l^{-1}$ ethidium bromide) at room temperature and after another 30 min, 1 ml of solution II was added (15 g $l^{-1}$ citric acid, 0.25 M sucrose and 40 mg $l^{-1}$ ethidium bromide). Until use, samples could be stored in the dark at 4°C for at least 1–2 weeks.

For the measurement of apoptotic cells, the Annexin-V-Fluos Kit from Boehringer Mannheim was used. The preparation, staining and measurement of the cells was carried out according to the manufacturer's instruction.

The cells were analysed in a FACStar+ cell sorter (Becton Dickinson), equipped with two argon ion lasers (Innova 90, Coherent). All dyes were excited with the 488-nm line. Ethidium bromide (EB) and propidium iodide (PI) fluorescence were detected at wavelengths above 590 nm. Fluorescein (FITC) fluorescence was detected by a bandpass filter at 530 nm. For each sample, 20 000 events were acquired. All parameters were recorded in logarithmic scale and the data were analysed with the Data Analysis System (DAS) software developed by Beisker (1994).

### Western blot analysis

Approximately 2 × $10^6$ cells were detached from the glass surface, washed with phosphate-buffered saline (PBS) and centrifuged. The pellet was resuspended in lysis buffer [Triton-X-100 1%, Tris 25 mM, sodium chloride 120 mM, phenylmethylsodiumazide (PMSF) 1 mM, Natriumorthovanidate 1 mM] and incubated on ice for 5 min. The lysate was clarified by centrifugation (14 000 *g*, 10 min, 4°C). Loading buffer [Tris-HCl pH 6.8 100 mM,

**Figure 1** Cell survival of HeLa cells after a 1-h cisplatin exposure, showing the development of drug resistance as a function of conditioning radiation treatment. –○–, Control; –▽–, two conditioning cycles (10× 2 Gy); –●–, three conditioning cycles (15× 2 Gy). Data for each curve represent one typical experiment (± s.d.)

**Table 1** Sensitivity of HeLa and HeLa-C3 cells to various drugs

| Cell lines | Doxorubicin | Etoposide | Vincristine | Cadmium chloride |
|---|---|---|---|---|
| HeLa | 0.022 ± 0.005 | 0.23 ± 0.04 | 0.0135 ± 0.0009 | 71.4 ± 6.5 |
| HeLa-C3 | 0.026[a] ± 0.002 | 0.21[a] ± 0.04 | 0.0015[b] ± 0.0004 | 76.9[a] ± 4.8 |

All figures are the $IC_{10}$ drug concentrations (μg $ml^{-1}$) necessary to reduce cell survival to 10% after continuous drug exposure as derived from survival. [a]Not significantly different for HeLa and HeLa-C3 cells. [b]$P < 0.01$.

© Cancer Research Campaign 1997 

**Figure 2** Radiation sensitivity of parental HeLa (-○-) and resistant HeLa-C3 cells (-●-). Data points are means (± s.d.) of at least three individual experiments

Dithiothreitol (DTT) 200 mM, sodium dodecyl sulphate (SDS) 4%, bromophenol blue 0.2%, glycerol 20%] was added to the supernatant and the samples were stored at – 18°C until use. The protein lysate was adjusted to the cell number. Total cellular protein was assayed according to the procedure of Lowry et al (1951), with bovine serum albumin serving as standard.

Before separation, the probes were heated at 90°C for 5 min, resolved on SDS–polyacrylamide gels (10–12%) and transferred on a polyvinyldifluoride (PVDF) membrane in TBS-T buffer (20 mM Tris, 137 mM sodium chloride, 1 M hydrochloric acid, 0.1% Tween 20). After blocking with 5% skimmed milk, the membranes were exposed to the primary antibody (1 μg ml$^{-1}$, ON). The filters were washed with TBS-T followed by incubation with the second antibody for 2 h at room temperature. The filters were washed again and then developed. The detection was accomplished with the enhanced chemiluminescence (ECL) method. The western blots were analysed using Pharmacia Biotech software Image Master 1D.

### RESULTS

After three cycles of fractionated γ-radiation treatment, HeLa cells developed a moderate cisplatin resistance ($R_f$ = 2.1), which had been stable for more than 150 cell generations (Figure 1). The drug-resistant HeLa-C3 cells exhibited similar doubling times as HeLa cells (20.5–23 h) and a similar protein content (175–195 μg 10$^{-6}$ cells). HeLa-C3 cells slightly changed their phenotype with more polyhedric morphology compared with the more elongated shape of the parental cells. As in murine cells after γ-irradiation, there was no cross-resistance to irradiation (Figure 2). Also, the sensitivity to doxorubicin and etoposide was unchanged, whereas the sensitivity to vincristine was enhanced (Table 1). Metallothioneins were unaltered, as evidenced by the identical toxicity to cadmium (an indirect measure of metallothioneins).

By flow cytometric cell cycle analysis, no differences between Hela and HeLa-C3 cells were found. The analysis of apoptotic cells with the Annexin-V-Fluos Kit was based on plasma membrane alterations occurring during the apoptotic process. Phosphatidylserine (PS) is translocated to the outer leaflet of the plasma membrane and can thus be detected by the fluorescein-labelled protein Annexin-V, which has a high affinity for PS. Figure 3A demonstrates the differences in the number of apoptotic

**Figure 3** Flow cytometric analysis of the number of apoptotic cells in HeLa and HeLa-C3 cells. (**A**) Apoptotic cells 48 h after cisplatin exposure (0.5 and 5.0 μg ml$^{-1}$ for 1 h) or after γ-irradiation (7.5 Gy). Mean value ±s.d. from three independent experiments with 6–8 single samples. (**B**) Time course of apoptotic cells 18, 24, 48 and 72 h after cisplatin exposure (5.0 μg ml$^{-1}$ for 1 h). Single experiment with triplicate data points. Apoptosis was assessed with the Annexin-V-fluos kit. □, Hela; ▨, Hela-C3

cells in both populations 48 h after cisplatin exposure (0.5 or 5.0 μg ml$^{-1}$ for 1 h) and 48 h after γ-irradiation (7.5 Gy). In the parental HeLa cells, there was a considerable increase in apoptotic cells from 4.8 ± 1.4% (control) to 27.1 ± 2.2% after the low and to 58.6 ± 7.3% after the high drug dose; in the resistant HeLa-C3 cells, this increase was significantly lower (7.5 ± 1.5% and 30.6 ± 10.6% respectively). A time-course study also revealed differences in the entry into apoptosis between both cell lines (Figure 3B). In Hela cells, the number of apoptotic cells increased continuously after cisplatin exposure and reached a plateau after

© Cancer Research Campaign 1997

**Figure 4** Expression of apoptosis-related proteins in HeLa and HeLa-C3 cells after cisplatin exposure. Western blot analysis was performed before (controls, □) and 24 h after a 1-h cisplatin exposure (5 μg ml$^{-1}$, ▨), using the following proteins: p53 (**A**), bcl-2 (**B**) and the 45-kDa ICE precursor protease (**C**). Mean relative chemiluminescence ± s.d. of three or more experiments

**Figure 5** Time course of protein expression in HeLa and HeLa-C3 cells after cisplatin exposure and 24 h after γ-irradiation. Western blots were prepared for HeLa (□) and HeLa-C3 cells (▨) after a 1-h cisplatin treatment (5 μg ml$^{-1}$) or after 7.5 Gy. The following proteins were analysed: p53, bcl-2, CPP32 and precursor ICE protease. Data are from one experiment (time course) or are mean relative values of different experiments ± s.d. (γ -irradiation)

48 h. In the resistant population, a significant increase in apoptotic cells was observed only 48 h after drug exposure. However, in HeLa-C3 cells, the variation was high and the increase after 18 h was not significant. After γ-irradiation, the number of apoptotic cells remained below 10% in both cell lines after 24 h (not shown) and 48 h (Figure 3A).

One of the main regulatory proteins involved in apoptosis and also associated with cisplatin resistance (Kastan et al, 1992; Tishler et al, 1993) is the p53 protein. Basal levels of this protein are low in HeLa and HeLa-C3 cells (Figure 4). Twenty-four hours after a 1-h cisplatin exposure, about threefold higher p53 protein levels were found in the resistant compared to the parental cells. The elevation of the p53 protein was observed between 16 and 30 h post drug exposure (Figure 5). At 48 h, the protein level had almost declined in both cell lines. After irradiation (24 h after 7.5 Gy), the suppressor protein was also expressed at a higher rate in HeLa-C3 cells. Among the p53-dependent apoptotic proteins, increased bcl-2 formation may be associated with drug resistance. As the expression of this anti-apoptotic protein is slightly reduced in HeLa-C3 compared with HeLa cells (Figure 4) and as differences in stability seem to be small (Figure 5), there is no indication of an involvement of this protein in the development of cisplatin resistance in HeLa-C3 cells.

Significant differences between HeLa and HeLa-C3 cells were demonstrated in the expression of ICE-related proteins. The formation of the catalytically inactive 45-kDa precursor ICE protein was reduced in HeLa-C3 cells and amounted to only about 35% of the basal level in HeLa cells (Figure 4). The precursor ICE-IgG did not cross-react with ICE p10 or p20 subunits. After drug exposure or after irradiation (Figure 5), these differences in

**Figure 6** Protein expression of CPP32 cysteine protease in HeLa and HeLa-C3 cells. Western blot analysis was performed in untreated controls, 24 h after a 1-h cisplatin exposure (5 μg $ml^{-1}$) or 24 h after exposure to 7.5 Gy. Mean relative chemiluminescence ± s.d. of three or more experiments protein expression persisted. Basal levels of the catalytically active 60-kDa heterotetramer were similar in both cell lines (relative chemiluminescence 9.0 for HeLa and 8.7 for HeLa-C3 cells). The protease CPP32, a member of the ICE family, displayed considerably lower basal protein levels in the resistant HeLa-C3 cells. After cisplatin treatment, CPP32 increased in both cell lines, but only about 45% of the protease level, measured in the parental cells, was attained in the resistant population (Figure 6). After irradiation (24 h after 7.5 Gy), the same expression of CPP32 protein was found in both cell lines.

### DISCUSSION

ICE-related proteases play a central role in the induction and execution of apoptosis. Their additional importance in the regulation of apoptosis was demonstrated in our study. Alteration of the expression of ICE-related proteases is associated with the development of cellular resistance in HeLa-C3 cells. CPP32, a member of the ICE family, which is expressed at a reduced rate in HeLa-C3 cells, is probably differentially affected or regulated by the type of insult or damage involved. The signalling pathway and/or the DNA cross-linking damage caused by cisplatin induces a lower CPP32 level in HeLa-C3 cells. Radiation damage that finally leads to single- and double-strand breaks results in similar CPP32 protein expression in HeLa and HeLa-C3 cells, in accordance with their identical cellular radiosensitivity. The induction and regulation of the apoptotic pathway is complex and only poorly understood. The growing family of ICE-related proteases are involved in the death-signalling pathways in an intricate pattern of cooperation and interaction. These proteases are characterized by structural homology and different, but overlapping, substrate specificity (Fernandes-Alnemri et al, 1994; Faucheau et al, 1995; Nicholson et al, 1995; Tewari et al, 1995*b*). Whether the various isoforms act in parallel or sequentially and how their signal is amplified – a process necessary for rapid apoptosis – is still unknown. Moreover, not all the activation processes that have been demonstrated in vitro may be relevant in vivo (Enari et al, 1995). In HeLa-C3 cells, lower basal levels of preICE (but not of the mature ICE) and reduced levels of CPP32 after cisplatin exposure (but not after irradiation) were demonstrated to be involved in the regulation of cisplatin resistance. However, there are probably far more proteases that are altered and that may affect the signalling pathway of cisplatin repair. CPP32 is synthesized as an inactive precursor; the mature protease is generated by autocatalysis or by other proteases, such as ICE (Fernandez-Alnemri et al, 1994; Tewari et al, 1995*b*).

CPP32 is supposed to act downstream of ICE, suggesting that reduced induction of CPP32 in HeLa-C3 cells may be associated with decreased activation due to the low precursor ICE protein levels in these cells. The mature protein can also be formed from truncated or mutant subunits, such as ICEε, which are efficiently transcribed, translated and folded and which act as dominant inhibitors of ICE activity (Wilson et al, 1994; Alnemri et al, 1995). If such mutant proteins are also formed under physiological conditions or in response to stress, they may alter or inactivate the apoptotic pathway, as in HeLa-C3 cells. Therefore, the unchanged expression of the mature 60-kDa ICE protein in HeLa-C3 cells does not provide evidence about the activity of the protein. A correlation of protein expression and enzyme activity in HeLa and HeLa-C3 cells will be the subject of further experimental study. Viral inhibitors of CPP32 have been described, such as the cytokine response modifier A (CrmA) (Miura et al, 1993; Tewari and Dixit, 1995) or the baculovirus gene product p35 (Bump et al, 1995). However, it is not known whether there are cellular homologues of these viral inhibitors of ICE-like proteases that might also be involved in the induction of cellular drug resistance in HeLa-C3 cells.

One of the possible substrates for CPP32 is PARP, an enzyme that is involved in DNA repair and genomic surveillance and integrity (Nicholson et al, 1995; Tewari et al, 1995*b*). At the onset of apoptosis, PARP is proteolytically cleaved, which highly activates an endonuclease implicated in internucleosomal DNA cleavage. In HeLa-C3 cells, the substrates are not known. It is speculated that the DNA-cross-linking damage, induced by cisplatin, could involve repair proteins, such as the high-mobility group protein (HMG) (Billings et al, 1992; Chow et al, 1995). These transcription factors function as activators/repressors of transcriptional regulation by enhanced binding to target promotor sequences. Overexpression of damage recognition proteins, also a possible substrate of ICE-related proteins, has been implicated in cisplatin resistance in HeLa cells (Chao et al, 1991).

Reduced expression of the ICE-related proteases correlates with reduced induction of apoptosis. After cisplatin exposure, the number of apoptotic cells is considerably lower in the drug-resistant than in the parental cells. There is evidence that the p53 suppressor protein affects the induction of apoptosis; however, only some, not all, apoptotic pathways are p53-dependent. Wild-type p53 function is essential for cellular response to DNA damage. The suppressor protein that is post-transcriptionally regulated may differ in protein stability and also in the expression of various mutant forms that functionally inactivate the protein. In HeLa-C3 cells, the p53 protein level is significantly elevated compared with the parental cells. In this study, it is not possible to differentiate between wild-type and mutant p53 suppressor protein, as the antibody used reacts with both types. Determination of the proportion of wild-type protein and of mutant p53 forms would give additional information on the involvement of this

© Cancer Research Campaign 1997

regulatory protein in radiation-induced alterations in HeLa-C3 cells. Moreover, there is no evidence to indicate whether the enhanced p53 protein expression after cisplatin exposure in HeLa-C3 is associated with alterations of ICE-related proteases. After cisplatin treatment, HeLa-C3 cells exhibit a much lower fraction of apoptotic cells along with an increased level of p53; after irradiation, p53 is again increased, but the number of apoptotic cells is low and similar in both cell lines. Whether and how p53 affects the induction of apoptosis and/or cisplatin resistance in HeLa cells is an open question. The above studies have been carried out in HeLa cells conditioned by three cycles of fractionated irradiation, which gives rise to an uncloned heterogeneous population. More detailed studies on the involvement of altered p53 in the regulation of cisplatin resistance in HeLa-C3 cells will now be initiated using single isolated clones to avoid interfering or overlapping effects.

One of the various p53-dependent proteins that is implicated in apoptosis is the bcl-2 protein. In HeLa-C3 cells, this protein does not seem to be associated with the regulation of cisplatin resistance. This anti-apoptotic protein remains at similar or slightly reduced levels in the resistant HeLa-C3 compared with the parental HeLa cells. Also, regulatory functions described for bcl-2 in the suppression of ICE proteins (Miura et al, 1993) may therefore not operate in cisplatin resistance in HeLa-C3 cells. However, bcl-2 is only one of several anti-apoptotic proteins in this death pathway, and it would be interesting to know whether other family members (such as bcl-$x_L$) are involved or whether the ratio of pro- and anti-apoptotic proteins is changed. Again, as mentioned above for the p53 data, modified protein expression of individual clones may be masked in these experiments.

Our present study was planned to investigate the development of cisplatin resistance of an uncloned human cell population after fractionated $\gamma$-irradiation and to determine which cell death pathways are altered. More detailed studies on the mechanisms of resistance will now be carried out with individual resistant clones.

In summary, we conclude that low-dose $\gamma$-irradiation induces moderate, but stable, cisplatin resistance in HeLa cells. Drug resistance is associated with increased levels of p53 protein and decreased levels of ICE-related proteases. As the response to radiation is unaltered both in terms of cell survival and CPP32 expression, the results suggest that CPP32 is involved in the signalling cascade of the death pathway initiated by cisplatin but not radiation damage.

### ACKNOWLEDGEMENT

The authors are grateful to Miss Claudia Jäger for excellent technical assistance.

### REFERENCES

Alnemri ES, Fernandes-Alnemri T and Litwack G (1995) Cloning and expression of four novel isoforms of human interleukin-1β converting enzyme with different apoptotic activities. *J Biol Chem* **270**: 4312–4317

Beisker W (1994) A new combined integral-light and slit scan data analysis system (DAS) for flow cytometry. *Computer Mth Programs Biomed* **42**: 15–26

Billings PC, Davis RJ, Engelsberg BN, Skov KA and Hughes EN (1992) Characterization of high mobility group protein binding to cisplatin-damaged DNA. *Biochem Biophys Res Commun* **188**: 1286–1294

Bump NB, Hackett M, Hugunin M, Seshagiri S, Brady K, Chen P, Ferenz C, Frandlin S, Ghayur T, Li P, Licari P, Mankovich J, Shi L, Greenberg AH, Miller LK and Wong WW (1995) Inhibition of ICE family proteases by Baculovirus antiapoptotic protein p35. *Science* **269**: 1885–1888

Chao CCk, Huang SL, Lee LY and Chao SL (1991) Identification of inducible damage recognition proteins that are overexpressed in HeLa cells resistant to cisplatin. *Biochem J* **277**: 875–878

Chen Y, Smith MR, Thirumalai K and Zychlinsky A (1996) A bacterial invasion induces macrophage apoptosis by binding directly to ICE. *EMBO J* **15**: 3853–3860

Chow CS, Barnes CM and Lippard SJ (1995) A single HMG domain in high-mobility group 1 protein binds to DNAs as small as 20 base pairs containing the major cisplatin adduct. *Biochemistry* **34**: 2956–2964

Eichholtz-Wirth H (1995*a*) Reversal of radiation-induced cisplatin resistance in murine fibrosarcoma cells by selective modulation of the cyclic GMP-dependent transduction pathway. *Br J Cancer* **72**: 287–292

Eichholtz-Wirth H (1995*b*) Restoration of cisplatin sensitivity by mild hyperthermia in radiation-induced cisplatin resistant mouse fibrosarcoma cells. *Int J Oncol* **7**: 935–939

Eichholtz-Wirth H and Hietel B (1994) Cisplatin resistance in mouse fibrosarcoma cells after low-dose irradiation *in vitro* and *in vivo*. *Br J Cancer* **70**: 579–584

Eichholtz-Wirth H, Reidel G and Hietel B (1993) Radiation-induced transient cisplatin resistance in murine fibrosarcoma cells associated with elevated metallothionein content. *Br J Cancer* **67**: 1001–1006

Enari M, Hug H and Nagata S (1995) Involvement of an ICE-like protease in Fas-mediated apoptosis. *Nature* **375**: 78–83

Faucheu C, Diu A, Chan AWE, Blanchet A-M, Miossec C, Hervé F, Collard-Dutilleul V, Gu Y, Aldape RA, Lippke JA, Rocher C, Su MS-S, Livingston DJ, Hercend T and Lalanne J-L (1995) A novel human protease similar to the interleukin-1β converting enzyme induces apoptosis in transfected cells. *EMBO J* **14**: 1914–1922

Fernandes-Alnemri T, Litwack G and Alnemri ES (1994) CPP32, a novel human apoptotic protein with homology to *caenorhabditis elegans* cell death protein Ced-3 and mammalian Interleukin-1β-converting enzyme. *J Biol Chem* **269**: 30761–30764

Kastan MB, Onyekwere O, Sidransky D, Vogelstein B and Craig RW (1992) Participation of p53 protein in the cellular response to DNA damage. *Cancer Res* **51**: 6304–6311

Lowry OH, Rosenbrough NJ, Farr AL and Randall RJ (1951) Protein measurement with the folin-phenol reagent. *Biol Chem* **193**: 263–275

Miura M, Zhu H, Rotello R, Hartwig EA and Yuan J (1993) Induction of apoptosis in fibroblasts by IL-1β-converting enzyme, a mammalian homolog of the *C. elegans* cell death gene *ced-3*. *Cell* **75**: 653–660

Nicholson DW, Ali A, Thornberry NA, Vaillancourt JP, Ding CK, Gallant M, Gareau Y, kGriffin PR, Labelle M, Lazebnik YA, Munday NA, Raju SM, Smulson ME, Yamin T-T, Yu VL and Miller DK (1995) Identification and inhibition of the ICE/CED-3 protease necessary for mammalian apoptosis. *Nature* **376**: 37–43

Nüsse M and Kramer J (1984) Flow cytometric analysis of micronuclei found in cells after irradiation. *Cytometry* **5**: 20–25

Tewari M and Dixit VM (1995) Fas- and tumor necrosis factor-induced apoptosis is inhibited by the poxvirus *CrmA* gene product. *J Biol Chem* **270**: 3255–3260

Tewari M, Beidler DR and Dixit VM (1995*a*) CrmA-inhibitable cleavage of the 70-kDa protein component of the U1 small nuclear ribonucleoprotein during Fas- and tumor necrosis factor-induced apoptosis. *J Biol Chem* **270**: 18738–18741

Tewari M, Quan ST, O'Rourde D, Desnoyers S, Zeng Z, Beidler DR, Poirier GG, Salvesen GS and Dixit VM (1995*b*) Yama/CPP32β, a mammalian homolog of CED-3, is a CrmA-inhibitable protease that cleaves the death substrate poly(ADP-ribose) polymerase. *Cell* **81**: 801–809

Thornberry NA, Bull HG, Calaycay JR, Chapman KT, Howard AD, Kostura MJ, Miller DK, Molineaux SM, Weidner JR, Aunins J, Ellison KO, Ayala JM, Casano FJ, Chin J, Ding GJ-F, Egger LA, Gaffney EP, Limjuco G, Palyha OC, Raju SM, Rolando AM, Salley JP, Yamin T-T, Lee TD, Shively JE, MacCross M, Mumford RA, Schmidt JA and Tocci MJ (1992) A novel heterodimeric cysteine protease is required for Interleukin-1β processing in monocytes. *Nature* **356**: 768–774

Tishler RB, Calderwood SK, Coleman CN and Price BD (1993) Increases in sequence specific DNA binding by *p53* following treatment with chemotherapeutic and DNA damaging agents. *Cancer Res* **53**: 2212–2216

Wang L, Bergeron L, Zhu H and Yuan J (1994) Ich-1, an Ice/ced3-related gene, encodes both positive and negative regulators of programmed cell death. *Cell* **78**: 739–750

Wilson DP, Black J-AF, Thomson JA, Kim EE, Griffith JP, Navia MA, Murcko MA, Chambers SP, Aldape RA, Raybuck SA and Livingston DJ (1994) Structure and mechanism of interleukin-1β converting enzyme. *Nature* **370**: 270–275

© Cancer Research Campaign 1997